# Effects of group communication norms on daily steps in a team-based financial incentive mobile phone intervention in Shanghai, China

**DOI:** 10.1186/s12966-025-01707-w

**Published:** 2025-01-18

**Authors:** Yingnan Jia, Yingcheng Xiao, Hao Chen, Klaus Gebel, Chengshu Li, Shuangyuan Sun, Qinping Yang, Siyuan Wang, Li Zhang, Jing Wang, Minna Cheng, Dantong Gu, Yan Shi, Ding Ding

**Affiliations:** 1 Key Lab of Public Health Safety of the Ministry of Education, School of Public Health, Fudan University, 130 Dongan Road, Shanghai, 200032 China; 2 Health Communication Institute, Fudan University, Shanghai, 200032 China; 3 School of Public Health, Faculty of Health, University of Technology Sydney, Sydney, NSW 2007 Australia; 4 Shanghai Municipal Center for Disease Control and Prevention, 1380 West Zhongshan Road, Shanghai, 200336 China; 5 Pudong New Area Center for Disease Control and Prevention, Shanghai, 200136 China; 6 Pudong New Area Center for Patriotic Sanitation Campaign and Health Promotion Counsel, Shanghai, 200129 China; 7 Institute of Otolaryngology, Clinical Research Center, Eye and ENT Hospital, Fudan University, Shanghai, 200031 China; 8 National Clinical Research Center for Aging and Medicine, Huashan Hospital, Fudan University, Shanghai, 200040 China; 9 Prevention Research Collaboration, Sydney School of Public Health, Faculty of Medicine and Health, The University of Sydney, Sydney, NSW 2006 Australia

**Keywords:** Physical activity, Walking, Financial incentive, M-health, Mobile phone intervention, Social norms

## Abstract

**Background:**

Mobile technology offers great potential for physical activity promotion, especially by facilitating online communication, however, the impact of group communication norms on intervention effectiveness remains unclear. This study aimed to evaluate the effect on daily steps of a team-based social norms-related intervention using a mobile application.

**Methods:**

The 13-week quasi-experimental study was conducted in Shanghai, China, from September to November 2019, involving 2,985 employees from 32 worksites. For the intervention group (n = 2,049), participants set a goal of 10,000 steps per day. The teams and individual members would receive points for meeting the daily goal, contributing to team-based rankings and financial rewards for the teams and their members. In addition, the intervention teams created dedicated WeChat groups to facilitate communication, which were also used to collect group chat messages. The communication type in these groups was classified into four types: (1) nudging – encouraging team members to be more active, (2) sharing – exchanging the completion of daily step goals, (3) feedback – providing responses or suggestions to team members, and (4) other -diverse topics that could not be classified otherwise. The control group only tracked their steps online.

**Results:**

The weekly average steps of the intervention group increased by 2,523 steps, while the control group increased by 470 steps. In the first 3 weeks of follow-up, the frequency of nudging of 7–18 times/week had a positive cumulative effect on the step counts. Sharing more than 3 times/week had a positive cumulative effect. Over 6 and 13 weeks of follow-up, nudging 19 times/week or more had a positive cumulative effect while sharing and feedback at any frequency negatively affected average weekly steps.

**Conclusions:**

Communication types within a team affected team-based step counts in a financial incentive intervention. The team-level social norms related to communications might have different cumulative effects on team-level physical activity. ‘nudging’ messages had a significant association with the change in individual-level step counts in the medium or long term.

**Trial registration:**

Pilot Project of the application of walking incentive technology in occupational groups, 2019, ChiCTR1900023813. Registered 13 June 2019, https://www.chictr.org.cn/showproj.html?proj=39858.

**Supplementary Information:**

The online version contains supplementary material available at 10.1186/s12966-025-01707-w.

## Background

Regular physical activity reduces the risk of type 2 diabetes, cardiovascular disease, certain cancers, and all-cause mortality [1–3]. Globally, every year, physical inactivity causes more than 5 million deaths [4] and INT$53.8 billion of health care expenditure [5]. In China, 63.5% of the working population engages in work with light occupation activity intensity, and only 6.4% of the working population participates in moderate-to-vigorous intensity leisure-time physical activity for 150 min or more per week [6]. Therefore, workplace interventions, currently recommended as one of the ‘eight best investments that work for physical activity’ [7], may be a suitable approach to promote physical activity in China [8].

Worldwide, walking is the most popular type of physical activity because it is accessible, requires no special skills or equipment, and is practically free [9]. Furthermore, the ubiquity of smartphones with built-in accelerometers has provided opportunities for physical activity monitoring and the evaluation of interventions [10, 11].

Of the intervention components that could be combined with smartphone tracking, financial incentive interventions have been gaining traction [12]. A recent systematic review and meta-analysis suggested that while financial incentives for physical activity have been proven to be effective, there are still areas for improvement [12]. Firstly, it is challenging to reach the least active with interventions. Studies have shown that workplace interventions generally have a participation rate of less than 50% and that older employees, those with a healthier BMI, and those who are less stressed and more physically active are more willing to participate in workplace physical activity interventions. Those employees who would need the intervention the most are the least likely to participate [13, 14]. One obstacle in getting inactive people active is the delayed health consequences. From a behavioural economics perspective, as the health benefits of physical activity are far in the future, providing immediate positive reinforcement may help people increase their level of physical activity [15, 16]. A meta-analysis of randomized controlled trials (RCTs) found that financial incentive interventions were effective in increasing goal achievement of physical activity during the intervention period [17].

To date, most internet-delivered physical activity interventions lacked interactions and social support, resulting in poor user engagement and retention [18]. Mobile health (mHealth) interventions have been defined as healthcare services or health promotion practices supported by mobile technology and devices, including mobile phone text messages, mobile phone calls, wearable or portable monitoring devices, mobile health applications, and telemedicine [19, 20].

Babcock et al. highlighted the importance of leveraging pre-existing social connections in mHealth interventions [21, 22]. Communication and messaging approaches may foster these connections in physical activity promotion. Effective communication not only reinforces participants' satisfaction with physical activity interventions but also helps to maintain engagement and accountability within a group [23]. In particular, messaging that provides feedback, social support, and reminders can enhance the effect of interventions by keeping participants motivated and informed [24]. Creating social norms, which refer to perceived expectations from others towards a given behaviour [25, 26], such as physical activity, may help improve the effect of financial incentive interventions. Specifically, injunctive norms refer to individuals’ perceptions of others’ approval of a given behaviour, while descriptive norms focus on individuals’ perceptions of the prevalence of others’ behaviour [27]. Social norms among users of running apps can lead to "herd behaviour," where individuals, through features like following and supporting others, are motivated to align their actions with their social networks [28]. Previous studies mainly focused on descriptive norm-based physical activity interventions among adolescents or office workers, however, the effect of social norm interventions has not been well established [29–31]. Furthermore, little is known about how objectively measured team-based social norms influence individuals’ physical activity in the context of financial incentives.

The current study is a team-based financial incentives step count intervention. The objectives of our study were to (1) examine the effect of an intervention to increase daily steps that used a combination of team and individual financial incentives; (2) explore how team-based social norms affect team members’ daily steps by analyzing team chat records.

## Methods

### Design and recruitment

**Fig. 1:**
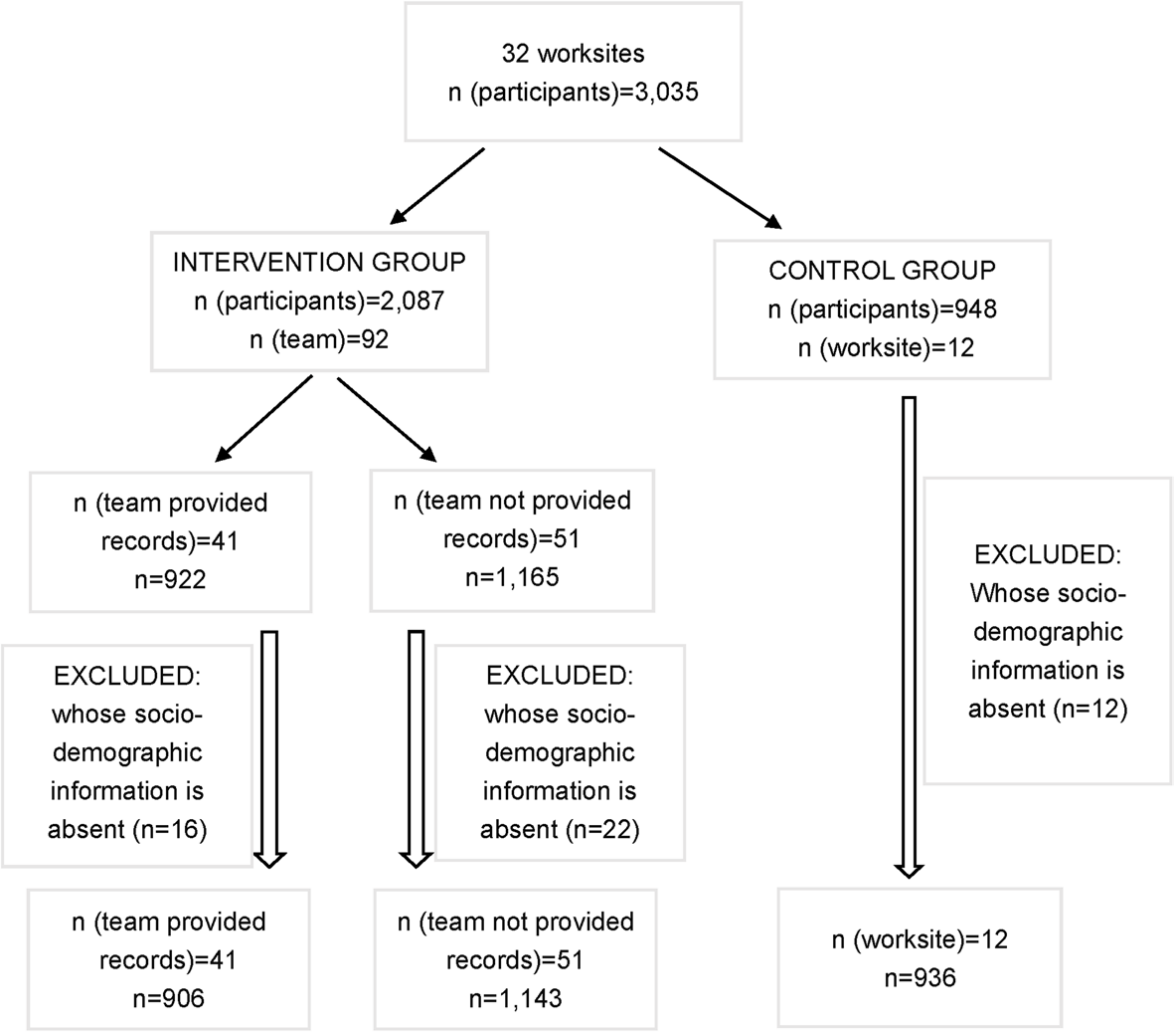
Flow chart

We conducted a 13-week non-randomized controlled trial step count intervention between September and November 2019. Firstly, based on purposive sampling, 32 work sites in Shanghai were recruited, from which 3,035 participants were invited. As this was a cluster-based trial, after informing participating teams about the financial incentive rules and specific requirements for the intervention, each worksite voluntarily opted for either the intervention or control group, with individuals from the same department allocated to the same team. Additionally, we conducted a clustering analysis, with the results presented in the supplementary file. A total of 92 teams (n = 2,087) for the intervention group were recruited from 20 worksites. The control group included 12 worksites (n = 948). Valid data were provided by 2,049 participants in the intervention group and 936 in the control group. The effective response rates were 98.2% and 98.7% respectively (Fig. 1). The inclusion criteria for teams and participants were: (1) at least 80% of the employees was set for each workplace, driven by strong leadership support and high employee willingness to participate, while ensuring representativeness; (2) employees had to be 18 years and above; (3) participants were required to have a smartphone with the function of tracking step counts. Exclusion criteria for participants were: (1) presence of heart disease, cerebrovascular disease, mental illness, or physical disorders; (2) pregnant women, as traditional Chinese cultural practices discourage physical activity among pregnant women, and there are widespread safety concerns regarding exercising during pregnancy, we excluded pregnant women from this study [32]; (3) employees who will leave the current workplace within the next few months. Unlike the intervention group, the control group consisted of individual participants rather than teams of participants. All participants provided written informed consent before taking part in the study.

### Intervention

Participants in the intervention group were instructed to form a team of 20–25 members and to elect a team captain. The choice of team size was primarily influenced by the typical number of employees within individual work departments, allowing for effective management and communication. Additionally, a larger team size helps foster greater inclusivity and engage diverse employees, including those who are less active. The intervention was developed based on the Theory of Reasoned Action and the Theory of Planned Behaviour, which emphasize the role of subjective and injunctive norms [25, 27, 33, 34]. We also applied the Behaviour Change Wheel (BCW) to guide intervention development. The BCW is a comprehensive and coherent framework that integrates behavioural theory to understand the specific behaviour change mechanisms within an intervention [35]. The BCW offers an effective approach for designing interventions that are tailored to the specific context and population, and it has been used to guide behaviour change intervention in the workplace [36]. The second layer of the BCW outlines a set of nine intervention functions (Education, Persuasion, Incentivisation, Coercion, Training, Enablement, Modelling, Environmental Restructuring, and Restrictions), which describe different ways an intervention can influence behaviour.

The intervention was designed with the concept that participants can be supported and nudged by team members. All participants were asked to complete a questionnaire, including sociodemographic characteristics. The online intervention was conducted by the CXA Group (/www.cxagroup.com/about/overview), an automated information technology platform that integrates wireless devices, enrollment processes, messaging, self-administered surveys, and automatic transfers of financial incentives. Daily step counts were measured by WeRun, a social fitness plugin for WeChat, the most widely used social media platform in China. The daily step counts of participants were obtained via a cloud-based secure server of CXA, which can synchronise their daily participation records with step counts. Before the start of the intervention, the team leaders were informed about the rules for scoring and awards. No other formal instruction or training was provided to the team leaders. Each leader and their team members worked together towards achieving the preset step count goals. The collaborative effort was primarily self-motivated, driven by the shared goal of accumulating points and improving team performance. Participants in the intervention group were given a goal of achieving at least 10,000 steps per day based on a health promotion program led by the Chinese Center for Disease Control and Prevention [37] and existing literature indicating that this target is associated with a reduced risk of all-cause mortality and incident cancer and cardiovascular diseases [38]. Participants in the control group were not provided with a specific goal as they continued with their usual activity levels. Additionally, 1–2 weekly tweets on physical activity knowledge and skills were posted on the WeChat official account. The platform delivered daily prompts to participants individually, to collect their step count as measured by a phone-based accelerometer and team members received points for confirming daily attendance and additional points for achieving the daily step count goal. The personal total score was the sum of daily points. The team score was the average of the individual total scores of all team members. The team score was ranked weekly, with the overall ranking determined after 13 weeks of intervention. Financial incentives included 3 components: (1) weekly team award: according to the weekly score of each team, and the top 50% of all teams received a financial reward of 200 RMB (100 RMB, equivalent to US$14.3 in 2019) every week. (2) Final team award: the top 50% of the teams based on overall ranking after 13 weeks received a financial reward of 3,000 RMB (US$429). (3) Individual incentives: Based on the total individual scores, the top 50 individual members received a financial reward of 200 RMB (US$28.6) (Table 1).

**Table 1:** Award and scoring rules for ranking both teams and individuals

Subjects	Award category	Scoring rule	Award rule
Intervention group only	Weekly team award	Based on the mean score across the individuals within the team	Top 50% of teams receive 200 RMB (US$28.6) weekly
	Final team award	Mean of individual scores within the team	Top 50% of teams receive 3,000 RMB (US$429) per team after 13 weeks
	Individual award	The sum of daily points from recording their attendance (10 points) and meeting the daily goal (90 points)	The top 50 individual participants receive 200 RMB (US$28.6)
Both intervention group and control group	Daily attendance award	Daily points from recording their attendance (10 points)	0.1 RMB per day (US$0.014)

The participants in the control group were only required to complete the basic sociodemographic questionnaire. To record the step counts in the control group, the platform also delivered daily prompts. Once the daily prompt was completed, participants in the control group received 0.1RMB (US$0.014) per day. There was no other intervention applied to the control group.

Table 2 illustrates how specific intervention measures were aligned with the key components of the TPB and BCW. It provides a clear overview of how each intervention element corresponds to the theoretical framework, ensuring that the intervention design is rooted in established behavioural theories.

**Table 2:** Correspondence of intervention components with the Theory of Planned Behaviour (TPB) and the Behaviour Change Wheel (BCW)

Intervention Components	TPB constructs	BCW constructs
Online sharing of physical activity-related knowledge and tips	Attitude	Education
Daily step count sharing and feedback	Perceived Behavioral Control	Modelling
Individual and team awards	Subjective Norm	Motivation, Incentivisation
Experience sharing	Behavioral Intention	Persuasion, Environmental Restructuring
Prompts, nudges and reminders within the team		

### Measures

#### Demographic variables

Demographic characteristics of the participants were collected by an online self-administered questionnaire, including date of birth, gender, marital status, and education.

#### Team-based social norms

During the 13-week intervention period, each team in the intervention group established a "WeChat" group for communicating and administration. Out of 92 teams, 41, consisting of 906 participants, provided the completed team chat records (3,268 valid records) for which a content analysis was conducted. Firstly, to develop an effective coding method and standard of classification, the researchers randomly selected the chat records of 8 of the 41 teams. Then, two researchers read all chat records independently to identify themes and categorized them into four communication types.

The final coding systems consisted of 4 types of communication:(1) nudging, (2) sharing, (3) feedback and (4) other. The 'other' category included messages that may not fit neatly into the first three types such as casual conversations that were unrelated to physical activity, as shown in Table 3. The coding of all the valid records was performed independently by the same two researchers in Microsoft Excel. Each record was coded by only one classification. Then the frequency of nudging, sharing and feedback were calculated for each team every week respectively.

**Table 3:** Codebook for team-based social norms related communication types

Communication themes	Definition	Examples	
Nudging	The records sent by team leaders or members, reminding others to clock in and achieve the daily goal	"There are still 5 team members who haven't achieved 10,000 steps. Come on!"	"@EveryoneWe still have to walk, we are slipping down the rankings every week, and we can't continue to fall behind. "
Sharing	The records sharing about daily step counts of their own or others		"I've walked 10,000 steps today."
Feedback	The records feeding back on the reward distribution, daily and weekly ranking of their team or other	"I heard that our team ranked 52^nd^ last week which was so close (to winning a team weekly step count award)."	"We got the rewards! During the first week, we were ranked 31st."
Other	Other information that did not fall into any of the three aforementioned types, such as health knowledge and information	"The cold front is coming and the temperature will drop tomorrow. Remember to put on more clothes when you take a walk."	"Just do your best and enjoy the exercise."

#### Step counts

Based on a pilot study conducted before the intervention, step counts according to WeRun correlated strongly (Spearman’s correlation coefficient 0.766, *p* < 0.001) with those measured by a hip-worn accelerometer (Actigraph GT3x-BT, Pensacola, FL, USA). Days with less than 1,000 were considered invalid and daily steps were truncated at 30,000 steps/day [39]. The primary outcome was mean daily valid steps during the intervention period (weeks 1–13). Baseline step levels were determined by calculating the average daily steps of participants over the two weeks prior to the intervention. The outcome variables were calculated based on team and individual levels, respectively.

### Data analysis

Demographics and baseline characteristics, such as age, gender, education level, marriage, and average daily valid steps, were summarized separately for intervention and control groups. We summarized the descriptive statistics of the continuous (mean, standard deviation), and categorical variables (number and proportions of participants in each category). Differences in characteristics between the groups were tested with Chi-square for categorical variables, and one-way ANOVA was used for normally distributed continuous variables. We used a distributed lag non-linear model (DLNM), including quasi-Poisson regression, with the following lag structures [multi-day lag (01–03), (01–07), (01–30)] to identify the cumulative effects of team-based social norms related to communication (nudging, sharing and feedback) on average steps per week. The DLNM model was as follows:

$$\text{log}[E\left({Y}_{t}\right)]=intercept+ns\left(week,3\right)+age+gender+cb({X}_{i},lag=13)$$

where: $cb$ is the cross-basis function, which simultaneously specifies the exposure–lag–response relationship in the exposure–response and lag–response dimensions. ${X}_{i}$ is nudging/sharing/feedback.

Due to the hierarchical structure of the data, we employed a two-level random-effect regression analysis to examine the associations between nudging, sharing, feedback and average steps per week, adjusting for age, gender, marriage status, education level, and baseline average steps per week. First, a null model was constructed to assess whether the average steps per week varied between groups by the intra-class correlation coefficient (ICC) index. The ICC was defined as the between-group variance divided by the within-group variance plus the between-group variance. Next, we examined the individual and group-level variables in two separate models to examine their effects on average steps per week. Finally, we used the random intercept and slope model, and by putting both individual and group-level variables in the model to examine their interaction effects. Alpha was set at 0.05 (two-sided). Data were stored in Microsoft SQL Server 2022. and statistical analyses were performed using R software version 4.1.2 (https://www.r-project.org/) with the “DLNM” and “lme4” packages.

## Results

### Descriptive statistics

Descriptive statistics of the individuals are presented in Table 4. Of the 2,985 participants in our study, 2,049 were in the intervention group and 936 in the control group. In the intervention group, participants were grouped into 92 teams according to their workplaces. In both the intervention (77.0%) and the control group (53.6%) there were more women than men. The intervention group was younger than the control group (mean = 35.8; SD = 11.0; mean = 40.1; SD = 21.4 years, respectively). The proportion of the participants who were married was 72.4% and 84.9% in the intervention and control groups respectively. The proportion of participants with a high education level (Bachelor and above) was lower in the intervention group (56.6%) than in the control group (68.3%). In the intervention group, 19.0% of the participants were healthcare workers versus 51.8% in the control group. Baseline weekly average steps were higher in the intervention group than in the control group (8,446 ± 4,126 vs 7,060 ± 3,210).

**Table 4:** Descriptive statistics of the study population

		Control (*n* = 936)	Intervention (*n* = 2,049)	***p***
**Gender (%)**	Male	215 (23.0)	950 (46.4)	< 0.001
	Female	721 (77.0)	1,099 (53.6)	
**Age (mean (SD))**		40.1 (21.4)	35.7 (11.0)	0.001
**Married (%)**	Yes	795 (84.9)	1,483 (72.4)	< 0.001
	No	141 (15.1)	566 (27.6)	
**Educational level (%)**	Junior high school and below	44 (4.7)	102 (5.0)	< 0.001
	High school / technical secondary school	71 (7.6)	351 (17.1)	
	Junior college	181 (19.3)	437 (21.3)	
	Bachelor	577 (61.6)	975 (47.6)	
	Master and above	63 (6.7)	184 (9.0)	
**Type of occupation (%)**	Services	37 (4.0)	430 (21.0)	< 0.001
	Health care	485 (51.8)	389 (19.0)	
	Production	0 (0.0)	770 (37.6)	
	Education	170 (18.2)	115 (5.6)	
	Civil service	244 (26.1)	345 (16.8)	
**Baseline weekly average steps (mean (SD))**		7,060 (3,210)	8,446 (4,126)	< 0.001

**Fig. 2:**
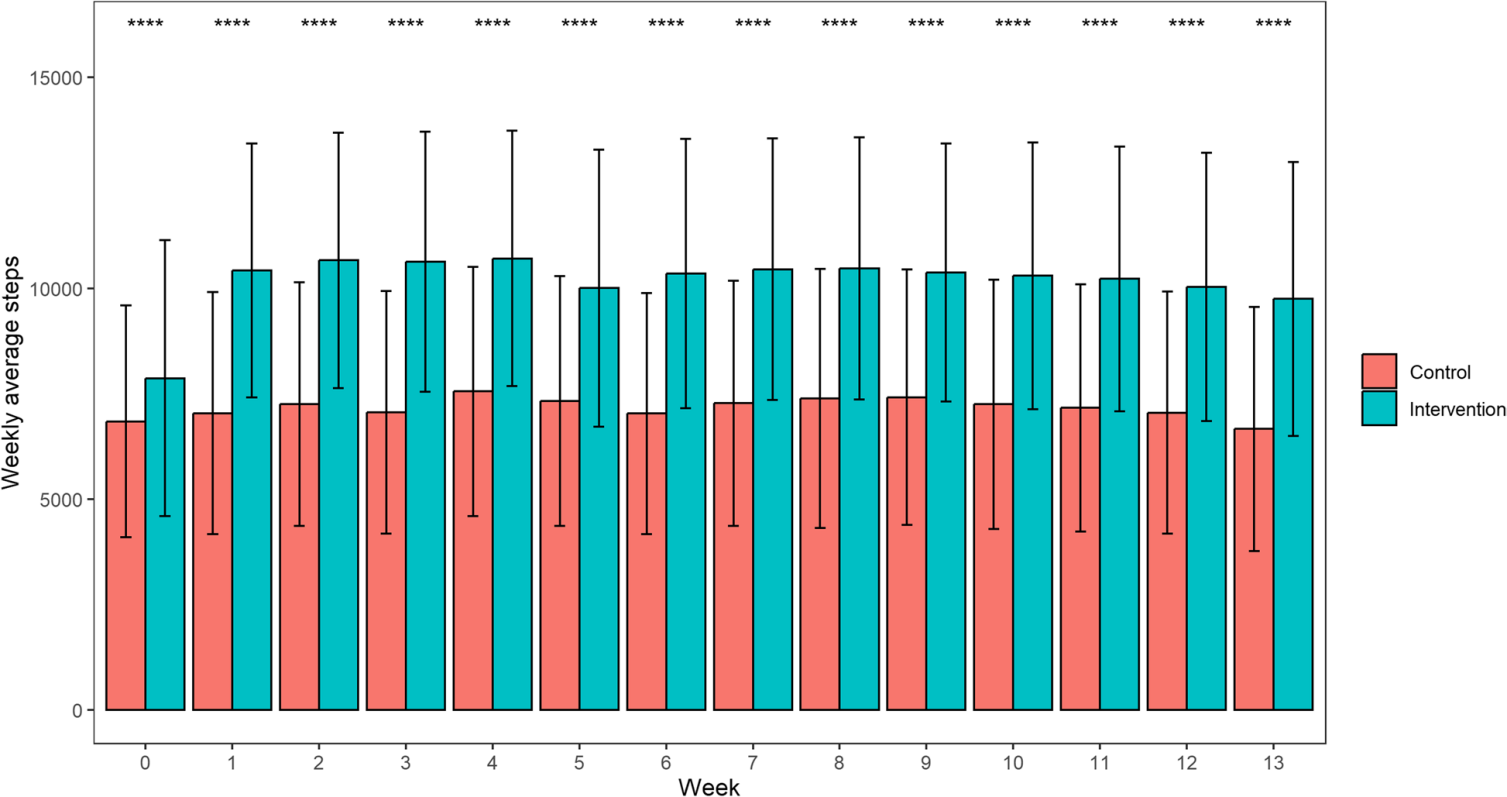
Weekly average steps between intervention and control group at baseline and follow-up

Figure 2 shows differences in weekly average steps between the intervention and the control group at baseline and follow-up. In the intervention group, compared to baseline, weekly average steps significantly increased at follow-up, but this trend was not seen in the control group.

### Intervention effects

**Fig. 3:**
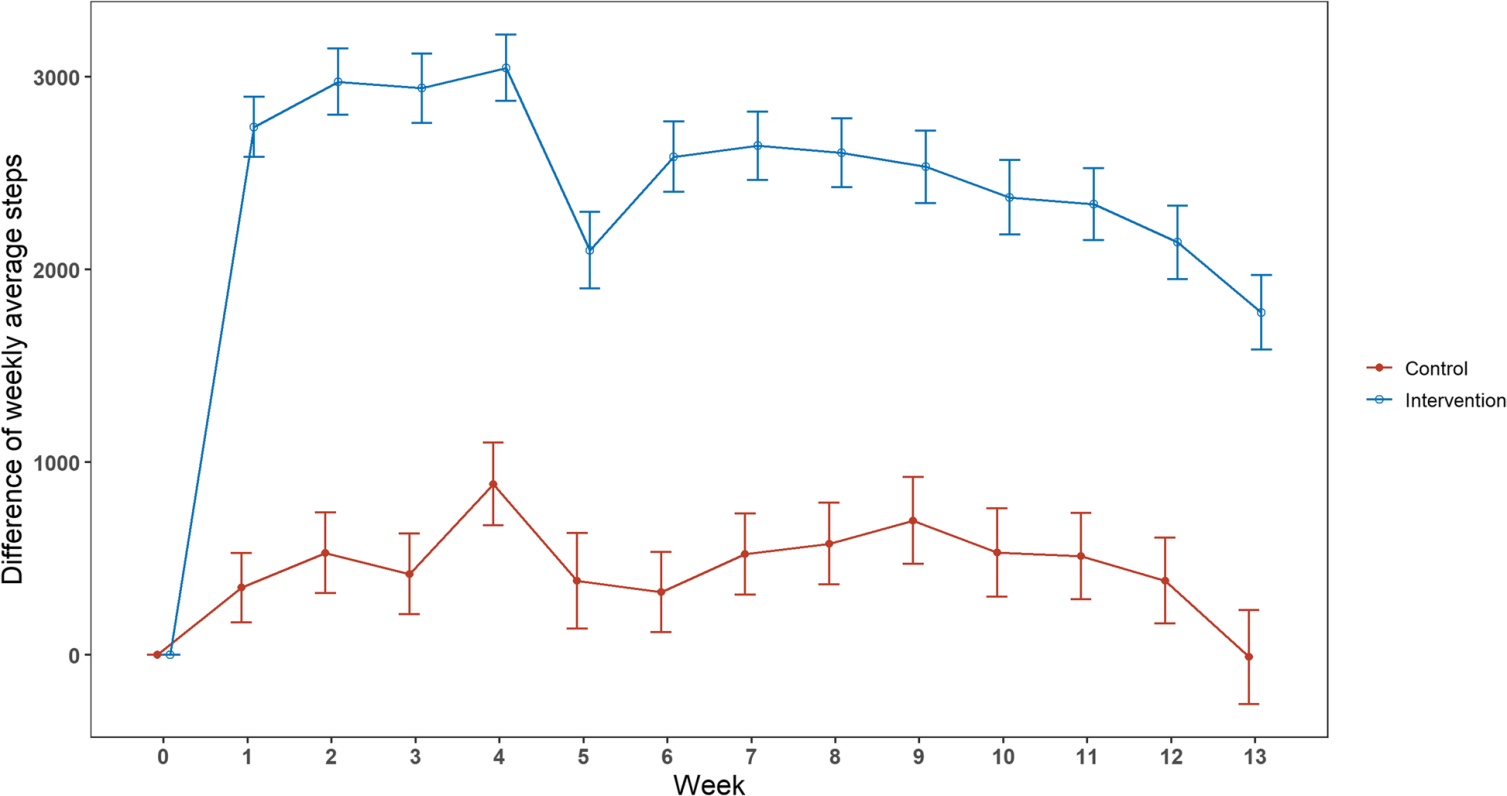
Differences in weekly average steps between intervention and control groups at baseline and follow-up

As shown in Fig. 3, at Week 1, the intervention group demonstrated a significant increase in weekly average steps from baseline (average increase = 2,740, 95%CI: 2,583 to 2,897), while the control group had an average increase of 349 steps (95%CI: 169 to 529). The intervention group had a peak in weekly average steps after 4 weeks, with an average increase of 3,046 steps (95%CI: 2,874 to 3,218). The control group also experienced a peak in weekly average steps after 4 weeks, but with a lower average increase of 887 steps (95%CI: 673 to 1,101). At the end of the last week of follow-up, the weekly average steps of the intervention group increased by 1,777 (95%CI: 1,584 to 1,971) while the weekly average steps of the control group decreased by 11 (95%CI: -255 to 232). Over the 13-week intervention period, the weekly average steps of the intervention group had an increase of 2,523, while the control group saw an increase of 470 steps.

### Team communication types and intervention effects

**Fig. 4:**
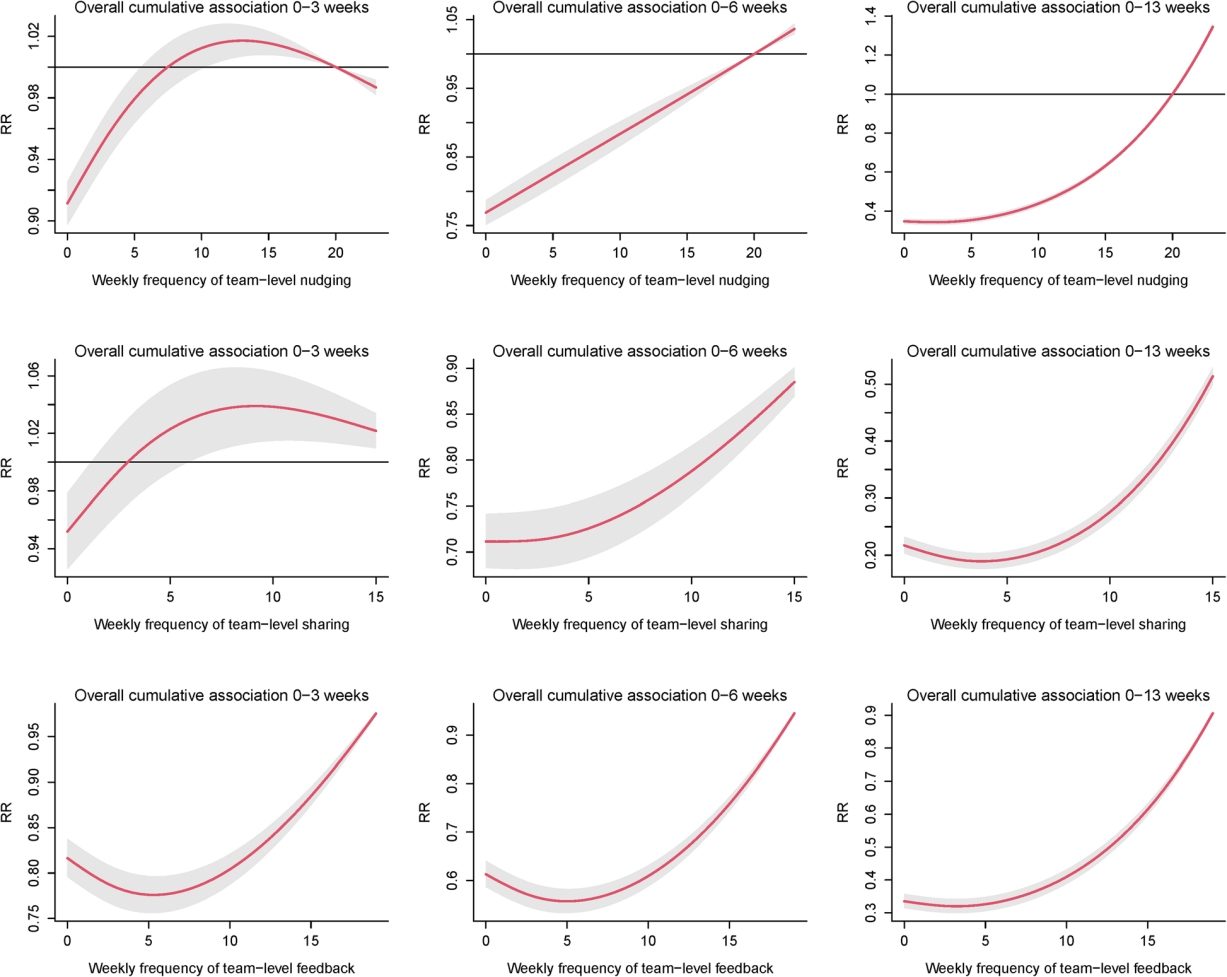
Cumulative effect analysis of team-based communication type variables in 41 intervention teams

Figure 4 shows the non-linear cumulative relationships between individual team-based communication types and average weekly steps. Findings suggested that the association between individual communication styles and step counts may differ at different stages of the intervention (3, 6, and 13 weeks). In the short-term (3 weeks of follow-up), the frequency of nudging of 7–18 times/week had a positive cumulative effect on the step counts. Sharing more than 3 times/week had a positive cumulative effect, while feedback at any frequency had a negative effect. Over 6 and 13 weeks of follow-up, nudging 19 times/week or more had a positive cumulative effect while sharing and feedback at any frequency negatively affected average weekly steps.

## Discussion

In this financial incentive intervention, we used 41,790 person-days of step counts and 3,268 chat records over 13 weeks to examine the effects of both team- and individual-based financial incentives on step counts and of team-level social norms as reflected by group chat history. Our study found that the financial incentive intervention was effective in increasing step counts. Interestingly, our findings suggest that team communication type that reflects the social norms of nudging, sharing and feedback might have different effects on team-level step counts. This raises the possibility that the communication within the team contributed to the intervention's success, alongside the financial incentives. The financial incentives may have been a primary motivator. Due to the team-based design of the intervention, team members spontaneously communicated to achieve group rewards. It is plausible that team communication and reinforcing behavioural norms played a crucial role in sustaining or amplifying the observed effects. Future studies could disentangle these components by testing interventions with and without financial incentives or focusing solely on the communication aspect of intervention to better understand the contribution of individual components. To our knowledge, this is the first study on the effects of team-level social norms with continuous measurements of both social norms related to communication and device-measured physical activity. These findings improve our understanding of team-based and financial incentive interventions for walking behaviours.

Over the 13-week intervention period, the weekly average steps of the intervention group increased by 2,523, while the control group increased by 470 steps. The effect of team-based financial incentives on step change was larger than in most other similar interventions. A systematic review and meta-analysis showed that in 12 of 23 included studies, financial incentives were associated with an average increase in daily steps of 607.1 during the intervention period (95% CI: 422.1 to 792.1; range from 93.0 to 3,907.0) [12]. Specifically, in an RCT from the USA on financial incentives to increase physical activity, the combined individual and team incentives led to an average of 1,446 more daily steps than in the control group [22]. A 24-week quasi-experimental study from Canada included 61,170 users and showed that adding team-based incentives increased mean daily steps by 537 [40]. We also found that the step counts first increased and then decreased during the 13 weeks of follow-up, which is consistent with other similar studies [22, 41]. This trend may be related to intervention fatigue, which means that the novelty of the intervention declines, which weakens its effect gradually.

Unfortunately, our intervention ended after 13 weeks and the evaluation of long-term sustainability was disabled by the subsequent COVID-19 outbreak and lockdown. Improving the sustainability of the intervention effect is a substantial challenge. Intrinsic motivation is often associated with the feelings of enjoyment, pleasure, or satisfaction [42], which may be fostered through adopting new habits of physical activity or obtaining goals. Furthermore, interventions like ours may also help establish a physical activity-friendly culture by changing social norms [43]. Weekly score rankings have been shown to enhance the effectiveness of physical activity interventions by motivating group members and sustaining improvements during follow-up periods [44]. Similarly, a recent intervention study among older women in Japan found that team-based social network incentives, which leverage social norms alongside financial incentives, were more effective than financial incentives alone. Notably, these effects persisted even after the intervention ended [45], suggesting that the presence of social norms may have a positive impact on participants' intrinsic motivation to engage in physical activity. The combination of stimulating individual intrinsic motivation and establishing team social norms may improve the sustainability of the intervention effect. Future studies should explore the long-term effects and sustainability of physical activity interventions. It is worth noting that our team-based financial incentive intervention involved weekly team rewards and final rewards for both teams and individuals. In addition, using insights from behavioural economics, weekly team rewards can help not only increase immediate feedback but also strengthen the relationship between team members [15, 16].

We explored how team-based social norms related to communication types affected team-level steps through a prospective study design. Analyses showed that non-linear cumulative relationships in different time lengths were also examined. For nudging, the frequency between 7–18 messages per week had a positive cumulative effect on team steps in the short-term (3 weeks of follow-up), while in the medium (6 weeks) and long term (13 weeks of follow-up), nudging 19 times or more showed a positive cumulative effect. Nudging from team members, family, friends and others can serve as external environmental factors as per BCW, influencing an individual's behavioural capacity, opportunities and motivation, and thus facilitating or impeding changes in physical activity behaviours [35]. For example, in a study that applied the BCW to improve the physical activity level of diabetes patients, text messages and nudging increased capacities and opportunities for physical activity [46]. For messages classified as ‘sharing’, more than 3 times a week had a positive cumulative effect in the short-term, while in the medium and long term sharing at any frequency had a negative cumulative effect on weekly average steps. Sharing and encouragement among teammates could influence an individual's perception of subjective norms and behavioural control, through which positive behaviour change could be engendered. Sharing, including discussions and conversations, not only fulfills individuals' social needs but also significantly influences group behaviours by shaping social norms and fostering unconscious behavioural mimicry. This social contagion effect underscores the importance of shared experiences in fostering a supportive environment for physical activity [47]. For example, compared to non-app users, app users demonstrated a stronger intention to maintain their running behaviour and more frequently encouraged other participants, likely due to the interactive features of the apps [28]. In terms of ‘feedback’ messages, whether in the short, medium or long term, any frequency had a negative cumulative effect. Evidence from a meta-analysis on the effects of feedback on health behaviours suggests that the impact of feedback on increasing physical activity is mixed, this may be due to different attributes of the feedback (e.g., frequency, personalisation, graphical versus text-based formats), which can have varying influences on the outcomes [48]. In some cases, feedback may provoke negative emotions that diminish motivation for healthy behaviours, potentially leading to disengagement. As shown in our study not all the feedback messages about team ranking results were positive, and less constructive feedback may have discouraged team members or led to them giving up on achieving the step goals subsequently. For messages classified as ‘nudging’, a stronger association with cumulative step counts was observed at higher frequencies. This may be because the goal of nudging is clearer than that of sharing and feedback, so it is easier to form subjective norms and herd behaviour. Our study did not find sharing and feedback to have a positive cumulative effect in the medium and long term. This result may be explained by insufficient follow-up time or insufficient frequency of sharing and feedback. Future studies should determine the potential dose–response effects of feedback and sharing on walking behaviour change. A significant contribution of this study was to provide the evidence that, within a financially incentivised physical activity intervention, different types of social norm-related communication within teams had varying effects on the intervention outcomes. Specifically, 'nudging' messages showed the strongest positive impact on improving the performance of the intervention.

## Strengths and limitations

Compared with existing studies, our study has several important strengths, such as continuously measured social norms related to communication, including messages of ‘nudging’, ‘feedback’ and ‘sharing’ nature by collecting chat records over 13 weeks of follow-up. Moreover, we examined the cumulative effects of social norms related to communication.

This study has some limitations. First, for practical reasons, randomisation of participants into intervention and control groups was not feasible in this quasi-experimental study. Instead, employers self-selected their participation into either the intervention or control group, which may have introduced selection bias and resulted in different demographic characteristics (e.g., gender) between the two groups. Although these demographic characteristics were adjusted for in the constructed model, such baseline differences may still lead to variations in responsiveness to the intervention, potentially impacting the estimation of the intervention effect. Second, this intervention provided a common step target for all participants. While 10,000 steps/day may be a suitable target for some, it may not be achievable for others, which may have limited its effectiveness in some participants. Future interventions should consider incorporating individualised goals. Third, the intervention period was only 13 weeks, which was shorter than other similar studies [29, 49]. We planned for longer-term data collection to examine the maintenance of the intervention effects post-intervention but were unfortunately unable to do so due to the subsequent COVID-19 pandemic and lockdown. However, despite the relatively short follow-up we managed to capture meaningful increases in step counts in the intervention group. Fourth, compared to a dedicated activity tracker, smartphones may lack accuracy due to various factors, such as the frequently and location of carrying the phone. Despite limitations, we considered using smartphones for step tracking a pragmatic approach because 1) it didn’t involve additional participate burden as all participants already used Wechat and Werun and therefore was likely to result in better adherence; and 2) if the sources of bias were random, the lack of accuracy may be less of a problem because intervention and control groups both used the smartphone tracker before, during and after the intervention. Additionally, the impact of the 'other' communication type on the effectiveness of physical activity interventions may have been overlooked in this study.

Finally, we did not include pregnant women in this study due to culturally specific safety considerations, despite physical activity being widely recommended to pregnant women internationally [50]. Because physical activity during pregnancy is discouraged according to traditional Chinese culture, we were concerned that including pregnant women could lead to safety concerns and negatively affecting the program’s reputation, thus undermining recruitment and engagement.

## Conclusions

This team-based financial incentive intervention significantly increased daily step counts. Based on objectively measured step counts and more than 3,000 chat records, our study revealed that the team-level social norms related to communications, including nudging, sharing and feedback, might have different cumulative effects on team-level physical activity. ‘nudging’ messages, but not the other two types of messages, had a significant association with the change in individual-level step counts in the medium or long term. Future interventions should capitalize on health-promoting social norms to maximize intervention effects.

## Supplementary Information

Supplementary Material 1.

## Data Availability

The datasets are available from the corresponding author upon reasonable request.

## Abbreviations

BMI: Body mass index
ANOVA: Analysis of variance
DLNM: Distributed lag non-linear model
ICC: Intra-class correlation coefficient
SD: Standard Deviation
COVID-19: Coronavirus disease-2019
BCW: Behaviour Change Wheel
